# A case of gastric duplication cyst in an 18-year-old female

**DOI:** 10.1016/j.ijscr.2025.111099

**Published:** 2025-02-28

**Authors:** Osama Hroub, Kareem Ibraheem, Mohammad Hroub, Ne’ma Manasrah, Abdalrahman N. Herbawi, Badawi Eltamimi

**Affiliations:** a Faculty of Medicine, Palestine Polytechnic University, Hebron 90200, Palestine; b Palestinian Clinical Research Center, Bethlehem, Palestine; c Gastroenterology Department, Al Ahli Hospital, Hebron 90200, Palestine

**Keywords:** Gastric duplication cysts, Endoscopic ultrasound (EUS), Endoscopic Unroofing, Gastric mass

## Abstract

**Introduction:**

Gastric duplication cysts (GDCs) are rare congenital anomalies, usually diagnosed in childhood, but can occasionally present in adults with non-specific symptoms such as abdominal pain, nausea, vomiting, and dysphagia. Advanced imaging, particularly endoscopic ultrasonography (EUS), plays a crucial role in diagnosis, while surgical or endoscopic resection is the definitive treatment.

**Presentation of case:**

An 18-year-old female with no significant medical history presented with recurrent epigastric pain radiating to the back, abdominal fullness, heartburn, and difficulty swallowing solid foods for one month. Upper endoscopy revealed a gastric mass, and EUS identified a 30 — 28 mm cystic lesion adjacent to the stomach fundus. Fine-needle aspiration confirmed the diagnosis of a gastric duplication cyst. The patient underwent successful endoscopic unroofing, leading to symptom resolution.

**Clinical discussion:**

GDCs in adults are uncommon and often present with vague gastrointestinal symptoms, making diagnosis challenging. Imaging modalities such as EUS and fine-needle aspiration are essential for differentiation from other gastric lesions, including gastrointestinal stromal tumors and pancreatic cysts. Traditional management involved surgical resection, but endoscopic approaches, such as unroofing, offer a less invasive alternative with favorable outcomes.

**Conclusion:**

This case emphasizes the need for GDCs to be considered in the differential diagnosis of gastric masses. Early identification with EUS and minimally invasive intervention, such as endoscopic unroofing, can effectively resolve symptoms and prevent complications.

## Highlights

• Rare Case, a gastric duplication cyst in an 18-year-old, typically seen in childhood.
• EUS with fine-needle aspiration confirmed the diagnosis.
• Minimally Invasive Treatment: Successful endoscopic unroofing led to symptom resolution.
• The role of EUS and endoscopy in diagnosing and managing GDCs

## 1 Introduction

Gastric duplication cysts (GDCs) are rare congenital anomalies primarily affecting children, with occasional cases in adults. Although they sporadically occur in the stomach, the ileum is the most prevalent location for duplications [1]. Only 4 % of gastrointestinal duplications involving the stomach [4]. When they are symptomatic, they typically show up as vomiting, upper abdominal pain, and in rare cases, a palpable abdominal mass. Surgical resection is curative, while cross-sectional imaging is diagnostic [1].

Even though it might be challenging to diagnose GDCs before surgery, some useful information has been obtained through new imaging methods. For GDCs, endoscopic ultrasonography (EUS) is an important diagnostic tool. The diagnosis of a GDC is more likely when EUS shows a cyst with an echogenic interior mucosal layer and a hypoechoic muscle layer [2].

It is challenging to make a comprehensive diagnosis of GDC, even with the assistance of diagnostic tools like endoscopy. The only definitive way to diagnose GDCs is through complete resection. This case study describes an uncommon instance of adult symptomatic GDC. The patient presented with abdominal fullness, heartburn, nausea, vomiting, weight loss, and difficulty swallowing, with mild substernal tenderness [2].

## 2 Case presentation

This case report follows the SCARE 2023 guidelines for surgical case reporting [12]. An 18-year-old female, otherwise healthy with no significant past medical history, presented to the emergency department in a private hospital with recurrent episodes of epigastric pain radiating to the back. The pain was not relieved by over-the-counter medications and was associated with abdominal fullness and heartburn that worsened when lying down but improved slightly when leaning forward. She also reported occasional nausea, vomiting, and difficulty swallowing solid food for one month. In addition, She reported gradual, unintentional weight loss over the past month. She denied any changes in bowel habits, fever, chills, or night sweats.

On clinical examination, the patient was hemodynamically stable and showed no signs of distress. Mild substernal tenderness was noted on deep palpation, while the rest of the examination was unremarkable. Laboratory studies revealed normal complete blood count (CBC) and liver and pancreatic enzyme levels.

Upper endoscopy revealed a subepithelial, well-circumscribed, non-ulcerated mass located in the gastric fundus along the greater curvature. Endoscopic ultrasonography (EUS) identified a 30 × 28 mm anechoic, thin-walled cystic lesion adjacent to the gastric wall, without evidence of solid components or septations.

**Fig. 1:**
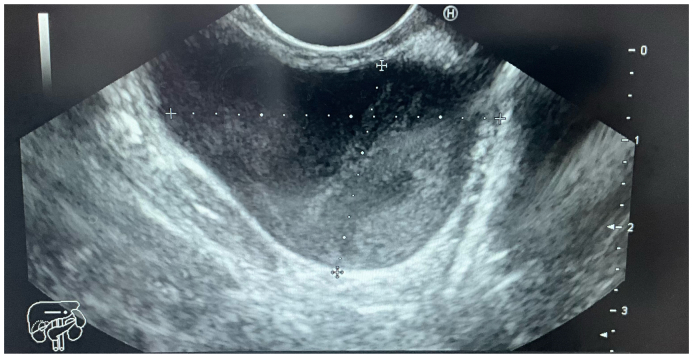
A regular mass can be seen at the great curvature of the stomach under gastroscopy.

**Fig. 2:**
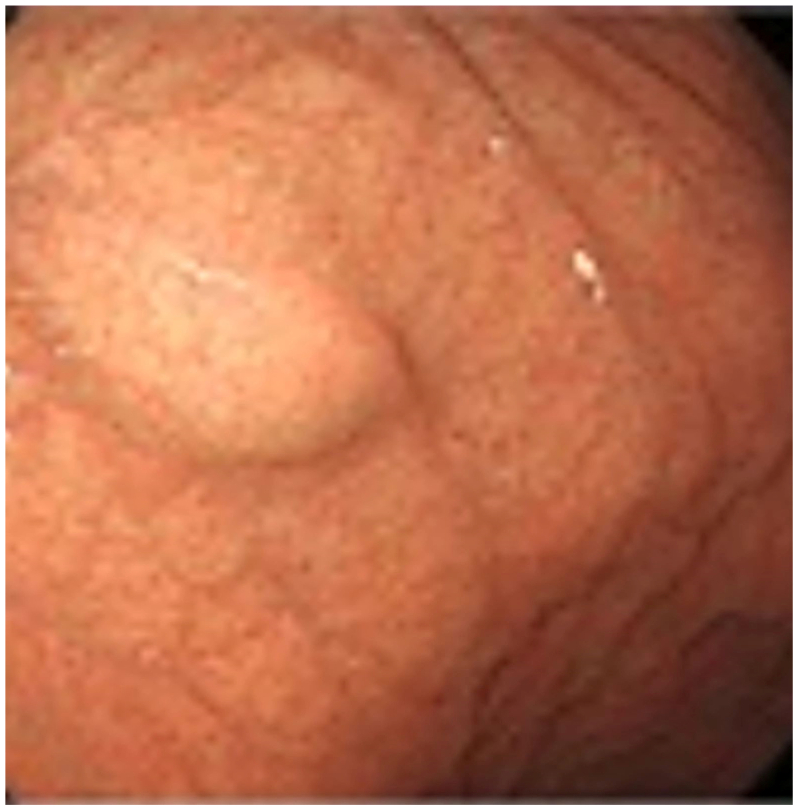
Endoscopic ultrasound (EUS) image. The lesion is seen to contain anechoic fluid, as well as a hyper-echoic structure. The lesion did undergo EUS-fine needle aspiration and revealed bronchogenic elements and mucus with a copious debris.

Upper endoscopy revealed a subepithelial, well-circumscribed, non-ulcerated mass located in the gastric fundus along the greater curvature (Fig. 1). The lesion appeared to be intramural, showing an elevated gastric mucosa. Endoscopic ultrasonography (EUS) identified a 30 × 28 mm anechoic, thin-walled cystic lesion adjacent to the gastric wall, without evidence of solid components or septations, suggesting an avascular lesion (Fig. 2). Fine-needle aspiration (FNA) of the cyst revealed highly viscous fluid with a positive string sign of 15 mm. Cytology demonstrated mucoid material with mucinophages, consistent with the cyst contents, and no malignant cells were identified. These findings confirmed the diagnosis of a gastric duplication cyst.

Based on the diagnosis, a plan was made for elective endoscopic unroofing of the cyst. The patient was informed about the diagnosis, the nature of the procedure, and its potential risks and benefits. She expressed understanding and consented to the proposed management. The procedure was performed under general anesthesia. After a standard gastroscopy to confirm the lesion location and size, the endoscopic mucosal resection technique was employed to create an access site over the cyst. The cyst wall was carefully incised, ensuring minimal disruption to the surrounding gastric mucosa. The contents of the cyst were aspirated, and the cyst was unroofed to allow drainage into the gastric lumen.

During the procedure, hemostasis was achieved with thermal coagulation, and the mucosal defect was left open to facilitate the natural drainage of the cyst. The patient was closely monitored post-procedure for any signs of complications, such as bleeding or perforation. She was discharged the following day with oral antibiotics and advised to follow up for a post-procedure endoscopic evaluation after 4 weeks. The cyst complete resolution was confirmed during the follow-up endoscopy, with no recurrence of symptoms.

## 3 Discussion

Gastrointestinal duplication cysts are rare congenital anomalies most commonly found in the ileum, followed by the esophagus, colon, jejunum, stomach, and duodenum. They occur in approximately 1 in 4500 live births and are present in 0.2 % of children [3]. However, there is no consensus regarding the distribution of gastrointestinal duplication cysts between genders [4].

GDCs are caused by the formation of a duplicate stomach during embryonic differentiation. Most are detected during prenatal screenings or in physical examinations of newborns and children, though some cases are diagnosed in adulthood. The mucosa of the duplicate stomach secretes gastric juice, leading to gradual cyst enlargement [5]. Gastric duplications are categorized into two forms: cystic (80 %) and tubular (20 %), with only 4 % of gastrointestinal duplications involving the stomach [4]. While there is no specific tumor marker for GDCs, markers like carcinoembryonic antigen (CEA) and carbohydrate antigen 19–9 (CA 19–9) have been investigated in clinical reports [13]. Accurate diagnosis is crucial, as only 35 % of GDCs are diagnosed preoperatively. These cysts can lead to complications such as obstruction, torsion, hemorrhage, or, in rare cases, left-sided portal hypertension or malignancy. Malignant pancreatic cystic tumors should also be considered in the differential diagnosis [7,10].

Gastric duplication cysts are typically diagnosed within the first year of life, with symptoms such as abdominal pain, vomiting, weight loss, a palpable abdominal mass, gastrointestinal obstruction, or perforation. Bleeding is rare, and only a few cases have been diagnosed in adulthood [6]. GDCs can cause a variety of non-specific symptoms, including abdominal and epigastric pain, nausea, vomiting, weight loss, bloody stools, and feeding difficulties. Due to their rarity and nonspecific presentation, GDCs are frequently misdiagnosed, often mistaken for gastrointestinal stromal tumors or other submucosal masses. This can delay diagnosis and appropriate intervention such as infection, ulceration, bleeding, perforation, fistula formation, and compression of nearby organs [4,10]. In the case presented, the patient reported recurrent episodes of epigastric pain radiating to the back, unrelieved by over-the-counter medications, and accompanied by a sensation of abdominal fullness.

GDCs may be discovered incidentally during screening endoscopy in asymptomatic patients. These cysts appear as submucosal masses compressing the gastric cavity, with a normal-appearing gastric mucosa. They are often difficult to distinguish from other submucosal lesions such as leiomyomas or gastrointestinal stromal tumors [7]. The pathology of GDCs remains controversial due to their rarity. Debates continue regarding presentation, imaging techniques, and diagnostic methods. A review of the literature shows a scarcity of GDC cases in adults, highlighting the need for further research in this area [7].

The diagnosis of GDCs requires precise radiological and morphological examinations. CT scans and endoscopic ultrasounds (EUS) are considered the most effective methods for diagnosis. Conventional radiography may reveal the cyst as an intramural filling defect or an outpouching on the greater curvature of the stomach. Ultrasound, a noninvasive imaging technique, typically shows the cyst as a hypoechoic lesion in the upper abdomen, often adjacent to the stomach, pancreas, liver, or biliary tracts. Cross-sectional imaging, such as CT or MRI, is critical in assessing the cyst's extent and its relationship to surrounding structures, though these methods may not always provide a definitive diagnosis [8].

CT scans typically show a well-defined, fluid-attenuation cystic lesion adjacent to or within the gastric wall, often along the greater curvature. The cyst wall may exhibit mild post-contrast enhancement, especially if lined with gastric mucosa, and complications such as infection or hemorrhage can increase its density. Calcifications, though rare, may be present in chronic cases [14]. EUS provides high-resolution imaging, showing a well-defined, anechoic lesion within the submucosal layer, often with a multi-layered reflecting the hyperechoic mucosa and hypoechoic smooth muscle layer. EUS is particularly useful in distinguishing GDCs from gastrointestinal stromal tumors (GISTs), pancreatic cystadenomas, or mesenteric cysts, guiding further management decisions [15]. GDCs can compress nearby organs, such as the pancreas, kidneys, spleen, and adrenal glands, leading to diagnostic confusion with lesions originating from these organs. They may also be mistaken for lymphangiomas, pancreatic pseudocysts, or mesenteric cysts [10].

While CT is commonly used, studies show it misclassifies GDCs as soft tissue masses in 43 % of cases. EUS is the most reliable diagnostic modality, as it allows detailed visualization of the lesion's layered structure, differentiating it from other gastric wall abnormalities [9]. EUS is particularly important for diagnosing gastrointestinal duplications by revealing the characteristic inner echogenic mucosal and outer hypoechoic muscle layers. EUS-guided fine-needle aspiration (FNA) can assist in ruling out malignancy, though its role remains controversial in definitive diagnosis [8]. Despite the availability of several imaging modalities, the optimal diagnostic method for GDC remains under discussion [9].

The treatment of gastric duplication cysts typically involves either endoscopic or surgical approaches. While medical therapies such as *H. pylori* eradication and proton pump inhibitors (PPIs) can manage symptoms associated with gastric duplication cysts (GDCs), they do not address the underlying lesion [5,9].Surgical methods include traditional laparotomy or minimally invasive procedures, such as endoscopic surgery. Options for surgical intervention include endoscopic fenestration of intragastric cysts and cyst dissection [5]. Unroofing may be just as successful as removing the cyst entirely, with the added benefit of preventing intestinal resection and allowing for an earlier start to oral feeding [16]. In this case, unroofing was favorable to avoid intensive mucosal injury, since the cyst was embedded deeply in the wall. Unroofing is an option, though it poses a risk of cyst fluid reaccumulation due to the remaining cystic wall. Recent advances in endoscopic surgery, such as endoscopic submucosal dissection (ESD), offer alternative methods for treating communicating ducts beneath the gastric mucosa [11] (Table 1; comparative studies). Laparotomy procedures may involve partial gastrectomy or gastric mucosal exfoliation. Laparoscopic resection of gastric duplications is becoming more common due to advancements in minimally invasive techniques [5]. Endoscopy-assisted laparoscopic surgery (EALS), a dual-mirror technique, is a promising new method for treating submucosal gastric tumors. It allows for more precise resection, less tissue damage, and faster recovery. EALS reduces exploration time and helps protect the gastric mucosa barrier, making it an increasingly preferred method for diagnosing and treating neonatal GDCs [5].

**Table 1:** Comparative clinical summary.

Feature	Case 1 (48 year-old woman) [17]	Case 2 (40 year-old woman) [11]
**Site**	Antrum	Pre-pyloric area
**Presentation**	Dyspepsia	Upper abdominal pain
**Duration**	Unknown	Unknown
**Imaging**	EUS: cystic lesion in the antrum 3 cm. CT scan: cystic lesion.	EUS an anechoic cyst in the submucosa. CT scan: cystic lesion with diameters of 3.2 × 1.2 × 1.1 cm.
**Endoscopy procedure**	endoscopic resection of the cystic submucosal lesion using a tunneling technique	endoscopic resection of the cystic submucosal lesion using a tunneling technique.
**Outcome**	Success lesion removal. No follow up data.	The patient did well and was discharged on the same day with no adverse events, and no recurrent symptoms were reported during the 12-month follow-up.

Complete excision is the preferred treatment for communicating GDCs to prevent complications such as obstruction, torsion, perforation, hemorrhage, and malignancy. While drainage and marsupialization are possible options, these procedures carry risks, such as exposing the cyst mucosa to gastric contents, leading to ulceration or malignant transformation. Therefore, drainage procedures like cyst jejunostomy are generally not recommended [10]. In most cases, surgery is the preferred treatment as it resolves symptoms and eliminates the risk of malignancy.

## 4 Conclusion

Gastric duplication cysts (GDCs) are rare congenital anomalies that can remain asymptomatic for years or present with non-specific gastrointestinal symptoms, leading to diagnostic challenges. This case highlights the importance of considering GDCs in the differential diagnosis of epigastric pain, particularly in young patients with unexplained symptoms. Endoscopic ultrasonography (EUS) plays a crucial role in the diagnosis, allowing for detailed visualization and differentiation from other submucosal gastric lesions. Endoscopic unroofing, as performed in this case, offers a minimally invasive alternative to surgical resection, reducing recovery time while effectively alleviating symptoms. This case adds to the growing body of literature supporting endoscopic techniques as a viable treatment for GDCs, emphasizing the need for further research to refine management strategies and long-term outcomes.

## CRediT authorship contribution statement

Kareem Ibraheem, Osama Hroub: Conceptualization, case analysis, manuscript writing, and editing.

Osama Hroub, Ne’ma Manasrah, Kareem Ibraheem: Data collection, literature review, and manuscript drafting.

Badawi Eltamimi, Mohammad Hroub, Abdalrahman N. Herbawi: Clinical management of the patient, data interpretation, and manuscript revision. All authors have read and approved the final manuscript and agree to be accountable for all aspects of the work.

## Patient consent

Written informed consent was obtained from the patient for the publication of this case report.

## Ethical approval

This case report is exempt from ethical approval in our institute, Palestine Polytechnic University.

## Guarantor

Badawi Eltamimi is the guarantor for this study, taking full responsibility for the research and its outcomes. Osama Hroub had access to all the data and made the final decision to publish the study.

## Funding statement

No sources of funding for this case report.

## Declaration of competing interest

The authors have no conflict of interest to declare.

## Data Availability

The data used to support the findings of this study are included in the article.
